# Long non-coding RNA PSMA3-AS1 promotes malignant phenotypes of esophageal cancer by modulating the miR-101/EZH2 axis as a ceRNA

**DOI:** 10.18632/aging.102716

**Published:** 2020-01-31

**Authors:** Bai-Quan Qiu, Xia-Hui Lin, Xu-Dong Ye, Wei Huang, Xu Pei, Dian Xiong, Xiang Long, Shu-Qiang Zhu, Feng Lu, Kun Lin, Xiao-Qiang Zhang, Jian-Jun Xu, Lu-Lu Sheng, Xue-Mei Zhang, Peng-Fei Zhang, Yong-Bing Wu

**Affiliations:** 1 Department of Cardiothoracic Surgery, The Second Affiliated Hospital of Nanchang University, Nanchang, Jiangxi, China; 2 Department of Oncology, Shanghai East Hospital, Tongji University School of Medicine, Shanghai, China; 3 Department of Oncology, Zhongshan Hospital, Fudan University, Shanghai, China; 4 Department of Emergency Medicine, Shanghai Jiao Tong University Affiliated Sixth People’s Hospital, Shanghai, China; 5 Department of Medical Oncology, Zhongshan Hospital, Fudan University, Shanghai, China

**Keywords:** long non-coding RNA, esophageal squamous cell carcinoma (ESCC), enhancer of zeste homolog 2, proliferation, metastasis

## Abstract

Backgrounds: Emerging evidences has demonstrated that dysregulation of long non-coding RNAs (lncRNAs) is critically involved in esophageal squamous cell carcinoma (ESCC) progression. However, the function of lncRNA PSMA3-AS1 in ESCC is unclear. Therefore, we aimed to explore the functions and potential mechanisms of PSMA3-AS1 in ESCC cells progression.

Results: Here, we found that PSMA3-AS1 expression was significantly up-regulated in ESCC tissues. Forced PSMA3-AS1 expression was correlated with tumor size, distant metastasis, and poor prognosis in ESCC patients. Functionally, PSMA3-AS1-overexpression promoted ESCC cells proliferation, invasion, and migration in vitro. Mechanistically, PSMA3-AS1 up-regulated EZH2 expression by competitively binding to miR-101.

Conclusion: PSMA3-AS1 is significantly up-regulated in ESCC tissues, and the PSMA3-AS1/miR-101/EZH2 axis plays a critical role in ESCC progression. Taken together, our results may provide promising targets for ESCC therapy.

Methods: PSMA3-AS1 and miR-101 expression were explored using qRT-PCR in ESCC tissues and cell lines. Immunohistochemistry assays were carried out to analyze EZH2 (enhancer of zeste homolog) protein expression. RIP, dual-luciferase reporter, fluorescence in situ hybridization, and biotin pull-down assays were used to detect the interactions of PSMA3-AS1, miR-101 and EZH2. The biological functions of PSMA3-AS1 in PSMA3-AS1-altered cells were explored using CCK-8, colony formation, wound healing, and transwell assays in vitro.

## INTRODUCTION

Esophageal squamous cell carcinoma (ESCC) is the fifth most common cancer and sixth leading cause of cancer-related deaths worldwide [1]. These dismal outcomes result from its notorious distant metastasis and recurrence [2]. The dysregulation of oncogenes and tumor suppressor genes plays important roles in the process of ESCC progression [3, 4]. Therefore, identifying new promising prognostic biological markers and investigating the detailed mechanisms underlying the progression of ESCC are urgently needed.

Long non-coding RNAs (lncRNAs, more than 200 nucleotides) are non-coding RNAs that participate in regulating the progression of several cancers, including ESCC [5–8]. Previous studies have shown that lncRNA proteasome subunit α3 antisense RNA 1 (PSMA3-AS1) acts as an oncogenic molecule in multiple myeloma [9]. However, the functions and mechanisms of PSMA3-AS1 in ESCC remain unclear. Recently, accumulating evidences have demonstrated that lncRNAs are involved in cancer progression as competing endogenous RNAs (ceRNAs) that sponge miRNAs to block their function, and then, up-regulate the downstream genes [6, 7]. For example, TUSC7 suppressed chemotherapy resistance in ESCC by downregulating miR-224 to modulate the DESC1/EGFR/AKT pathway [10]. Forced TTN-AS1 expression promotes ESCC cell proliferation and metastasis via sponging miR-133b [11]. Whether miRNAs are regulated by PSMA3-AS1 via competing endogenous (ceRNA) mechanisms also needs further investigation. Here, we demonstrated that 1) PSMA3-AS1 expression is higher in ESCC tissues than in adjacent non-tumor tissues; 2) higher PSMA3-AS1 indicated a poor prognosis in ESCC patients; and 3) PSMA3-AS1 sponges miR-101 to promote the proliferation, invasion, and metastasis of ESCC via up-regulating enhancer of zeste homolog 2 (EZH2) expression. Taken together, our results confirmed the sponging role of PSMA3-AS1/miR-101 in ESCC progression, and might thus provide promising prognostic and therapeutic targets for ESCC.

## RESULTS

### PSMA3-AS1 is up-regulated in tumor tissues and high expression indicates poor prognosis in esophageal cancer

**Figure 1:**
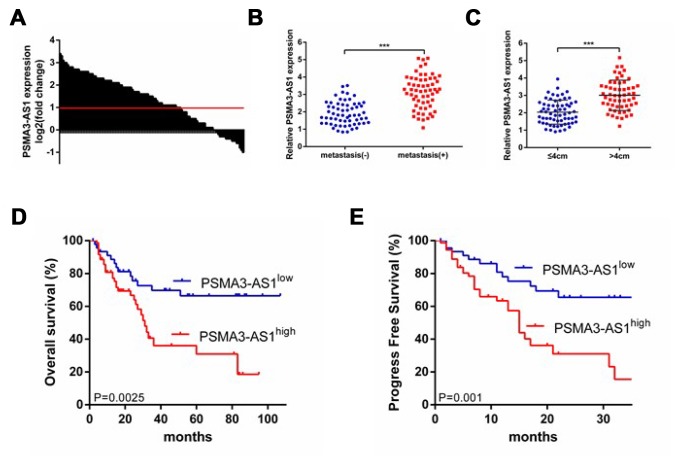
**PSMA3-AS1 is overexpressed in ESCC tissues and higher PSMA3-AS1 expression is correlated with a poor prognosis for ESCC.** (**A**) PSMA3-AS1 expression in ESCC tissues and their corresponding adjacent non-tumor tissues according to RT-qPCR analysis. GAPDH was used as an internal control for loading. (**B**) In total, 120 patients were divided into groups with and without distant metastasis. The diagram shows PSMA3-AS1 expression in each group. ***p < 0.001. (**C**) In total, 120 patients were divided into ≤ 4 cm and > 4 cm size groups. The diagram shows PSMA3-AS1 expression in each group. ***p < 0.01. (**D**) and (**E**) The OS and cumulative recurrence rates of 120 patients with ESCC were compared between the PSMA3-AS1^low^ and PSMA3-AS1^high^ groups using the Kaplan–Meier method (log-rank test). The data are represented as the mean ± SD, n=3. ***P < 0.001.

First, we investigated the expression patterns of PSMA3-AS1 between esophageal tumor tissues and paired adjacent non-tumor tissues using RT-qPCR analysis. The results showed that 61.7% (74/120)of tumor tissues exhibited significantly increased PSMA3-AS1 expression compared with paired adjacent non-tumor tissues (Figure 1A). Moreover, increased PSMA3-AS1 expression was positively correlated with distant metastasis (Figure 1B) and larger tumor sizes (Figure 1C). To further explore whether high PSMA3-AS1 expression was associated with poor prognosis, we divided the patients into PSMA3-AS1^low^ and PSMA3-AS1^high^ groups according to their PSMA3-AS1 expression levels. Kaplan–Meier analysis revealed a significantly longer median OS in patients with low PSMA3-AS1 was compared with those with high PSMA3-AS1 (Figure 1D). In addition, patients with higher PSMA3-AS1 expression had significantly shorter PFS than patients with lower PSMA3-AS1 expression (Figure 1E). Multivariate analysis revealed that a high PSMA3-AS1 expression status was an independent indicator for poor prognosis in esophageal cancer patients (Tables 1 and 2). Collectively, these results indicate that PSMA3-AS1 might be a useful prognostic biomarker and a promising therapeutic target in esophageal cancer.

**Table 1:** Correlation between PSMA3-AS1 and clinicopathological characteristics in 120 HEC patients.

	**Variables**	**No. of patients**	**PSMA3-AS1 expression level**			**low**	**high**	***P***
			Age					0.707
	<60	60	22	38	
	≥60	60	24	36	
Gender					0.113
	Male	70	31	39	
	Female	50	15	35	
Tumor stage					0.000
	I–II	55	32	23	
	III–IV	65	14	51	
Distant metastasis					0.000
	Yes	59	5	54	
	No	61	41	20	
Tumor size					0.000
	≤4 cm	64	34	30	
	>4 cm	56	12	44	
Differentiation					0.000
	Well/moderate	60	34	26	
	Poor	60	12	48	

*P values <0 .05 were considered statistically significant. The Pearson Chi- square test was used.

**Table 2:** Univariate and multivariate analyses of factors associated with overall survival

**Factors**	**Univariate, P**	**Overall survival**		
		**Multivariate**
			**HR**	**95%CI**	**P value**
Sex (female vs. male)	0.425				NA
Age (<60 vs. ≥60)	0.174			NA
Tumor size (≤4cm vs. >4cm)	0.253			NA
Metastasis (Yes vs. No)	0.027			NA
Tumor stage (I/II vs. III/IV)	0.124			NA
Differentiation (Well/moderate vs. poor)	0.001	0.521	0.272-0.998	0.049
PSMA3-AS1 expression (high vs. low)	0.000	0.398	0.194-0.816	0.012

OS overall survival, NA not adopted, 95%CI 95% confidence interval, HR hazard ratio, Cox proportional hazards regression model

### Up-regulation of PSMA3-AS1 promoted the growth and migration of esophageal cancer cells

**Figure 2:**
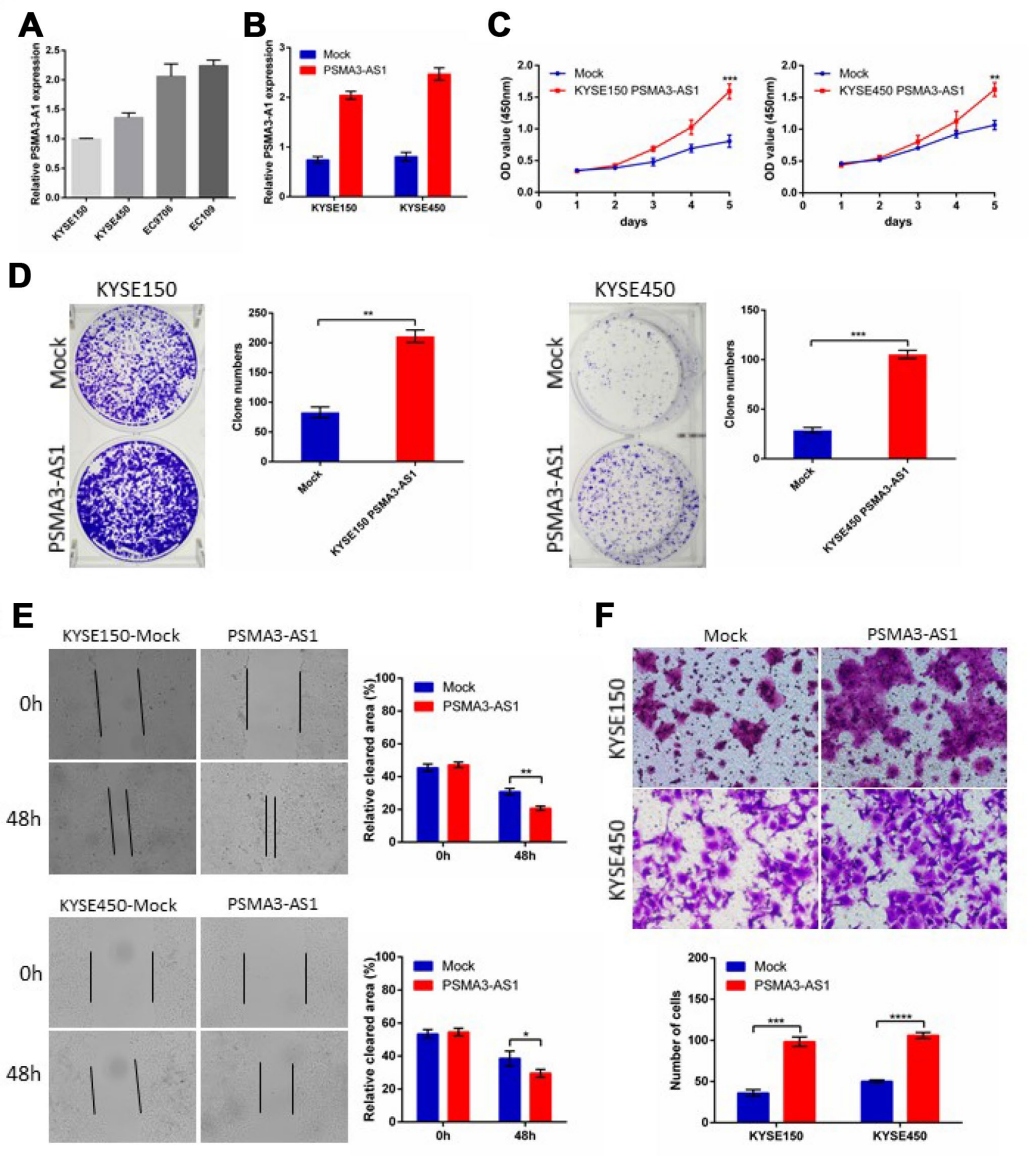
**Forced PSMA3-AS1 expression promotes ESCC cell proliferation, migration, and invasion in vitro.** (**A**) PSMA3-AS1 expression in several ESCC cell lines was examined using RT-qPCR analysis. GAPDH was used as an internal loading control. (**B**) PSMA3-AS1 expression in ESCC KYSE150 and KYSE450 cells was modified by cDNA transfection. (**C**) and (**D**) Cancer cell proliferation was measured using CCK-8 (**C**) and clone formation assays (**D**). (**E**) Cancer cell migration was measured using wound healing assay. (**F**) Cancer cell invasion was measured using transwell assay. The data are represented as the mean ± SD, n=3. *p < 0.05, **p < 0.01, ***p < 0.001, ****p < 0.0001.

PSMA3-AS1 expression is up-regulated and positively correlated with tumor size and metastasis in esophageal cancer patients. To further explore the biological roles of PSMA3-AS1 in esophageal cancer cells, we established stable PSMA3-AS1-overexpressing cell lines via lentiviral infection in KYSE150 and KYSE450 cell lines (which have low PSMA3-AS1 expression) and validated the up-regulation of PSMA3-AS1 by RT-qPCR (Figure 2A and 2B). CCK-8 and colony formation assays showed thatPSMA3-AS1 overexpression significantly promoted the proliferation of esophageal cancer cells (Figure 2C and 2D). Next, to explore the role of PSMA3-AS1 in esophageal cancer cell migration, we performed wound healing and transwell migration assays. Compared with mock cells, esophageal cancer cells overexpressing PSMA3-AS1 had significantly increased wound healing and cell migration (Figure 2E and 2F).

### PSMA3-AS1 knockdown suppressed proliferation and migration in esophageal cancer cells

**Figure 3:**
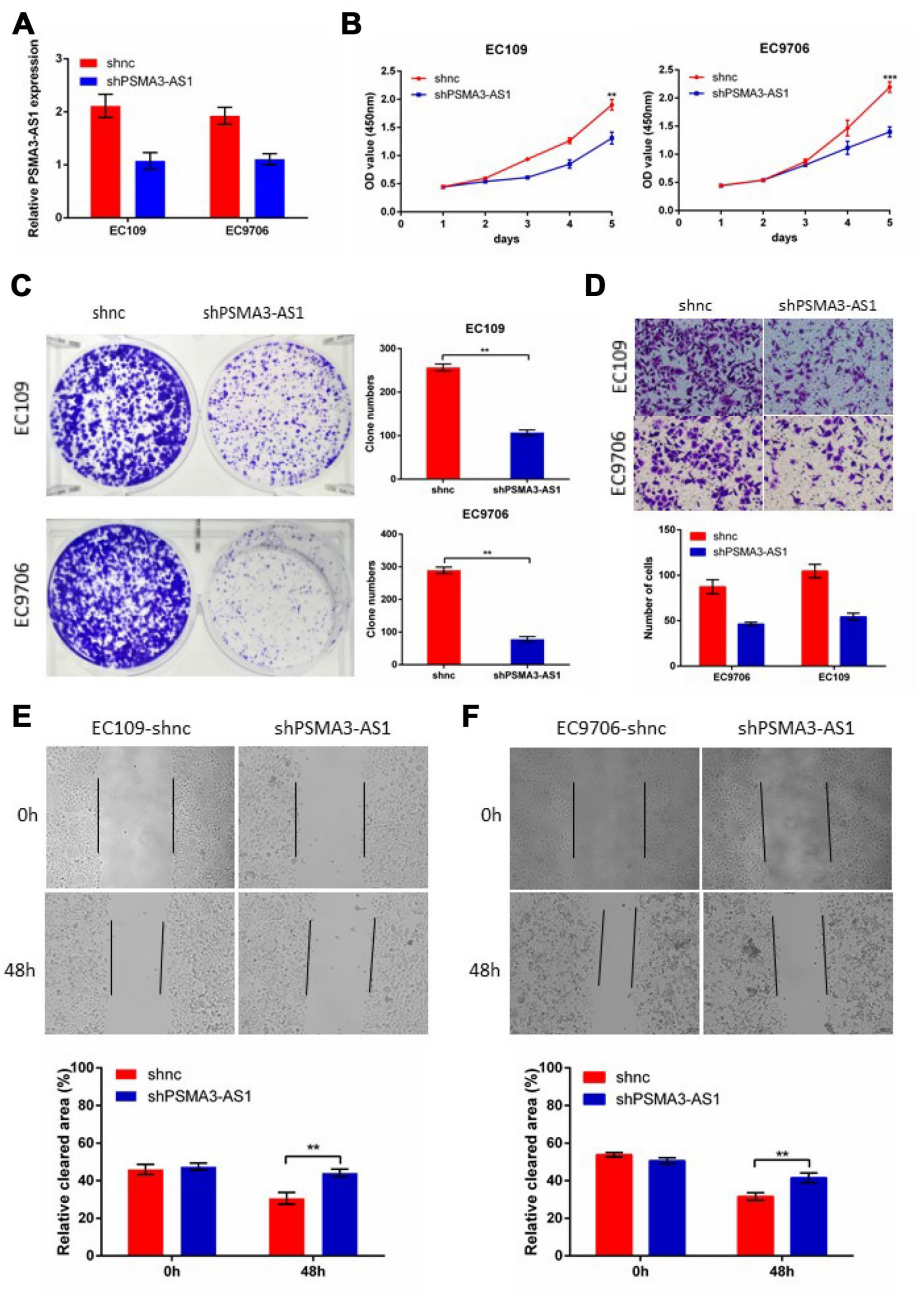
**Decreased PSMA3-AS1 expression inhibits ESCC cell proliferation, migration, and invasion in vitro.** (**A**) PSMA3-AS1 expression in ESCC EC9706 and EC109 cells was modified by shRNA transfection. (**B**) and (**C**) Cancer cell proliferation was measured using CCK-8 (**B**) and clone formation assays (**C**). (**D**) Cancer cell invasion was measured using transwell assay. (**E**) Cancer cell migration was measured by using wound healing assay. The data are represented as the mean ± SD, n=3. *p < 0.05, **p < 0.01, ***p < 0.001.

Next, to further verify the biological roles of PSMA3-AS1in esophageal cancer cells, we established stable PSMA3-AS1 knockdown via lentiviral infection in EC9706 and EC109 cell lines (which have high PSMA3-AS1 expression) and validated the down-regulation of PSMA3-AS1 by RT-qPCR (Figure 3A). CCK-8, colony formation, wound healing and transwell assays were then performed to monitor the proliferation and migration function of esophageal cancer cells in response to PSMA3-AS1 knockdown. The results showed that PSMA3-AS1 knockdown specifically suppressed the proliferation and migration of EC9706 and EC109 cells (which have high PSMA3-AS1 expression) in vitro (Figure 3B–3E).

### PSMA3-AS1 functions as a ceRNA and negatively modulates miR-101 expression

**Figure 4:**
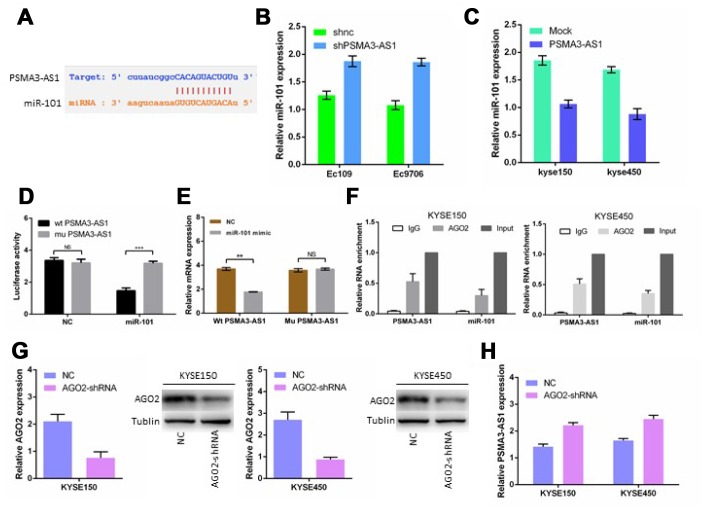
**PSMA3-AS1 functions as a ceRNA for miR-101 in ESCC cells.** (**A**) Target sequences in PSMA3-AS1 predicted to bind to miR-101. (**B**) and (**C**) miR-101 expression after forced/decreased PSMA3-AS1 expression was detected in ESCC cells by RT-qPCR. (**D**) Wild-type or mutated PSMA3-AS1 was transfected into HEK-293T cells with miR-101 or a negative control. Luciferase activity was detected 48 h after transfection. (**E**) RT-qPCR showed PSMA3-AS1 levels in the streptavidin-captured fractions from KYSE-150 cell lysate after transfection with biotinylated miR-101 or negative control (NC). (**F**) RIP experiments were performed using an antibody against AGO2 in extracts from KYSE-150 cells. (**G**) AGO2 expression in ESCC KYSE150 and KYSE450 cells was modified by shRNA or cDNA transfection. (**H**) TPSMA3-AS1 expression after decreased AGO2 expression was detected in ESCC cells by RT-qPCR. The data are represented as the mean ± SD, n = 3. *p < 0.05; **p < 0.01.

Previous studies have shown that lncRNAs usually function as ceRNAs and participate in the regulation of other mRNAs by competing for shared miRNAs [6, 7, 12]. A total of 164 miRNAs, including miR-101, were predicted as candidate targets of PSMA3-AS1 using starBase V3.0, (Figure 4A). Next, we found that PSMA3-AS1 knockdown significantly up-regulated miR-101 expression in esophageal cancer cells, whereas PSMA3-AS1 overexpression reduced miR-101 expression (Figure 4B and 4C). Furthermore, the luciferase reporter assay results demonstrated that forced expression of miR-101 significantly decreased the luciferase activity of the wild-type (wt) PSMA3-AS1 vector rather than the mutant (mu) PSMA3-AS1 vector (Figure 4D). Moreover, PSMA3-AS1 was pulled down by biotinylated miR-101, whereas mutagenesis of the binding sites for PSMA3-AS1 in miR-101 abolished this interaction (Figure 4E). AGO2 is a vital components of the RNA-induced silencing complex (RISC) and functions as a key regulator of miRNA function [13]. The levels of PSMA3-AS1 and miR-101 were highly enriched in esophageal cancer KYSE150 and KYSE450 cells as suggested by a RIP assay using an AGO2 antibody (Figure 4F). Furthermore, AGO2 knockdown increased PSMA3-AS1 expression in esophageal cancer KYSE150 and KYSE450 cells (Figure 4G and 4H). Thus, our data suggest that PSMA3-AS1 functions as a miR-101 sponge in esophageal cancer cells.

### PSMA3-AS1 restores the expression of EZH2 by binding to miR-101

**Figure 5:**
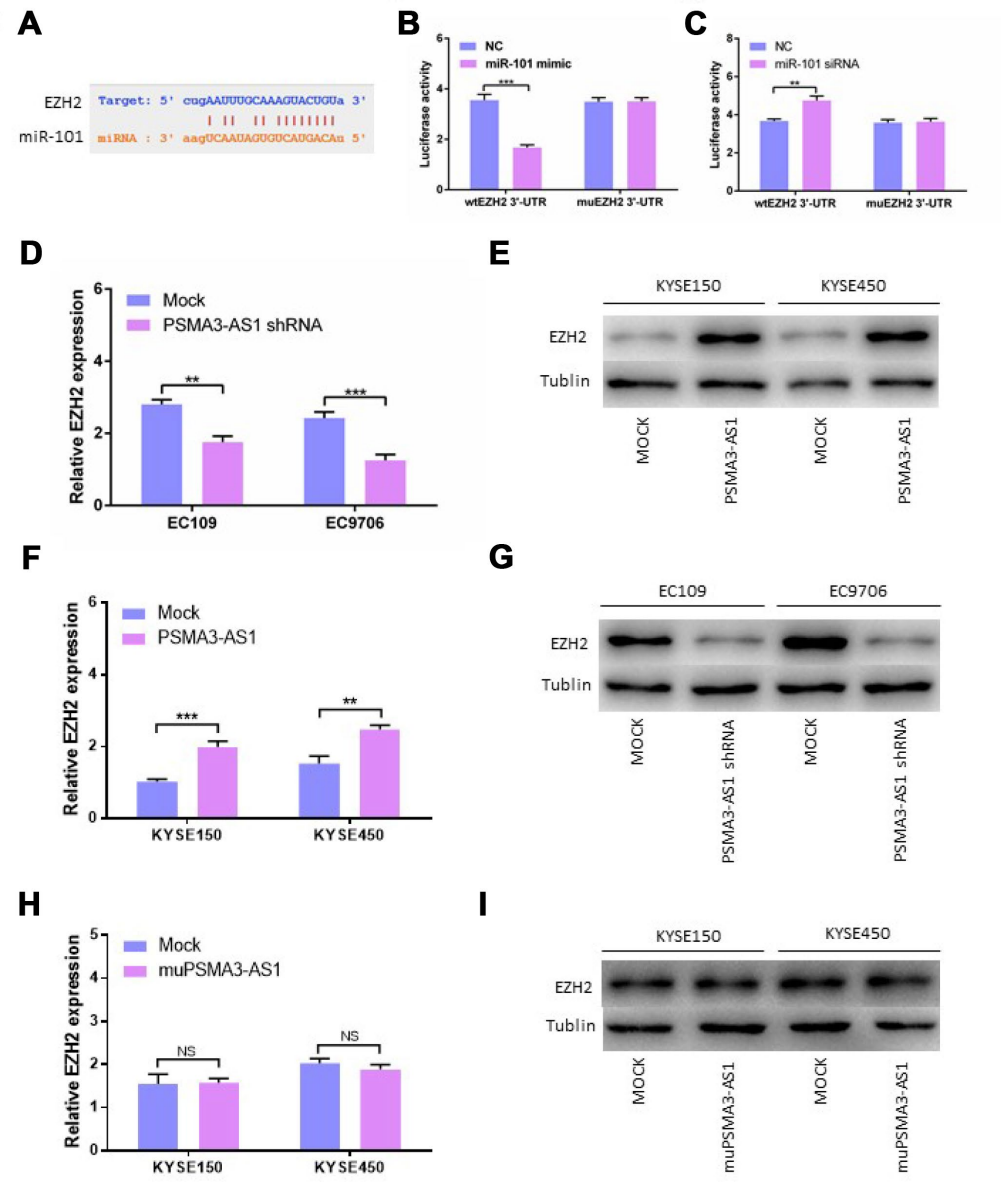
**PSMA3-AS1 restores EZH2 expression by binding to miR-101.** (**A**) Target sequences in the EZH2 3′-UTR predicted to bind to miR-101. (**B**) Wild-type or mutated 3′-UTRwas transfected into HEK-293T cells with miR-101 or a negative control. Luciferase activity was detected 48 h after transfection. (**C**) Wild-type or mutated 3′-UTRwas transfected into HEK-293T cells with miR-101 siRNA or a negative control. Luciferase activity was detected 48 h after transfection. (**D**) and (**E**) EZH2 expression was detected after PSMA3-AS1 expression upregulation in ESCC cells by RT-qPCR and western blotting. (**F**) and (**G**) EZH2 expression was detected after PSMA3-AS1 expression donwnregulation in ESCC cells by RT-qPCR and western blotting. (**H**) and (**I**) EZH2 expressions was detected after mutant PSMA3-AS1 expression upregulation in ESCC cells by RT-qPCR and western blotting. The data are represented as the mean ± SD, n = 3. *p < 0.05; **p < 0.01.

Using StarBase 3.0, we confirmed that there is one binding site for miR-101 in the EZH2 3′-UTR. Next, we constructed luciferase reporter vectors containing the wild-type (wt) or mutant (mu) EZH2 3′-UTR (Figure 5A). When miR-101 mimics were transfected, the luciferase activity of the WT reporter was reduced significantly in esophageal cancer cells (Figure 5B). The luciferase activity of the WT reporter was increased by transfecting miR-101 siRNA in contrast to transfection with miR-101 mimic (Figure 5C). Consistent with our expectations, the luciferase activity of the mu reporter did not change in response to miR-101 mimic or siRNA transfection (Figure 5B and 5C). Moreover, down-regulation of PSMA3-AS1 significantly inhibited the expression of EZH2 compared with the mock cells (Figure 5D and 5E). However, up-regulation of PSMA3-AS1 significantly increased the expression of EZH2 in esophageal cancer cells(Figure 5F and 5G). However, the results showed that up-regulation of mutant PSMA3-AS1 did not increase the expression of EZH2 in esophageal cancer cells (Figure 5H and 5I). All of the above results indicated that PSMA3-AS1, miR-101 and EZH2 acted together via a ceRNA mechanism

### EZH2 knockout altered PSMA3-AS1-induced proliferation and migration in esophageal cancer cells

**Figure 6:**
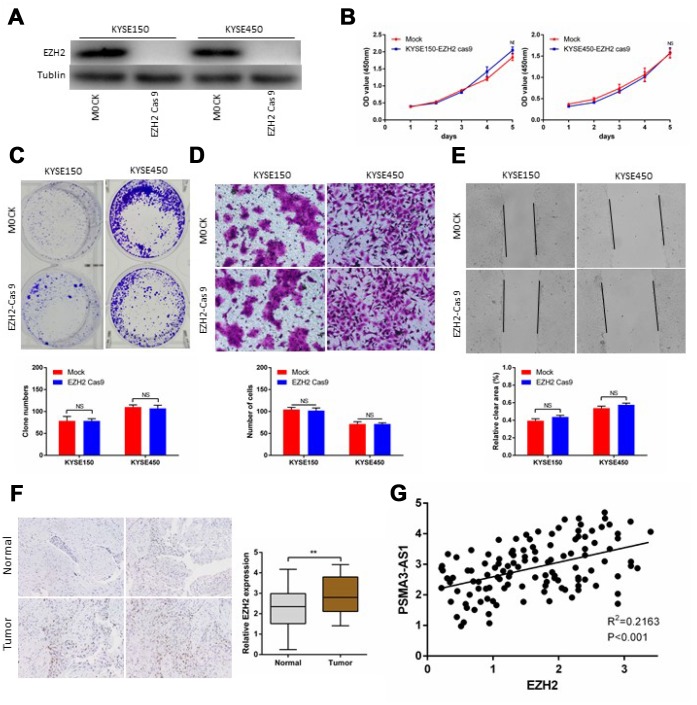
**PSMA3-AS1-induced esophageal cancer cells progression in an EZH2 dependent manner.** (**A**) EZH2 expression in ESCC KYSE150 and KYSE450 cells was modified by CRISPR/Cas9 gene editing. (**B**) and (**C**) Cancer cell proliferation was measured using CCK-8 (**B**) and clone formation assays (**C**). (**D**) Cancer cell invasion was measured by transwell assay. (**E**) Cancer cell migration was measured by wound healing assay. (**F**) Representative ESCC cases in the tissue microarray were analyzed by immunohistochemical staining for EZH2. (**G**). A positive correlation between PSMA3-AS1 and the number of EZH2-positive cells was observed in ESCC tissues (R^2^ = 0.2163; P < 0.001). The data are represented as the mean ± SD, n=3. *p < 0.05, **p < 0.01, ***p < 0.001.

Next, we successfully performed CRISPR/Cas9 gene editing of EZH2 in KYSE150 and KYSE450cells as confirmed by a significant reduction in EZH2 protein expression (Figure 6A). CCK-8 and colony formation assays showed that PSMA3-AS1 overexpression did not affect the proliferation of esophageal cancer cells with EZH2 knocked out (Figure 6B and 6C). According to wound healing and transwell migration assays, wounding healing and cell migration were not increased in ESCC EZH2-knock out cells overexpressing compared to negative control cells (Figure 6D and 6E). Next, we examined the expression of EZH2 in120 ESCC samples. The results showed that 67.5% (81/120) of the tumor tissues exhibited significantly increased expression of EZH2 compared with paired adjacent non-tumor tissues (Figure 6F). Moreover, increased EZH2 expression was positively correlated with increased PSMA3-AS1 expression (Figure 6G).

## DISCUSSION

In recent decades, increasing studies have reported abnormal expression profiles of lncRNAs in a series of cancers [6, 14]. Recent investigations have shown that dysregulated lncRNAs play critical roles in modulating tumor progression. Nonetheless, the molecular mechanisms by which lncRNAs participate in cancer progression remain elusive. Currently, only a few lncRNAs have been confirmed to participate in ESCC progression. In this study, we report that PSMA3-AS1 exerts critical roles in the progression of ESCC. At high levels, PSMA3-AS1 can bind directly to and act as a sponge for miR-101, which downregulates the expression of miR-101. Moreover, clinical pathological characteristics illustrated that increased expression of PSMA3-AS1 was positively associated with distant metastasis, larger tumor sizes, and worse prognosis for ESCC patients.

Recently, increasing studies have reported the relationship between lncRNAs dysregulation and ESCC progression from a clinical perspective and explored the molecular mechanisms of oncogene or tumor suppressor genes in the clinic. For example, CASC9 was significantly upregulated in ESCC tissues compared with non-tumor tissues and increased CASC9 expression was associated with worse prognosis and distant metastasis in ESCC patients [15]. LUCAT1 expression was significantly increased in ESCC tissues compared with adjacent non-tumor tissues. Moreover, patients with high LUCAT1 expression had a worse prognosis than patients with low LUCAT1 expression [16]. LncRNAs are critical members of ceRNA networks and have been reported to have multiple biological functions, including acting as miRNA sponges that participated in regulating the lncRNA/miRNA/mRNAaxis in several tumors [6, 7, 17].

Cell proliferation and distance metastasis are critical in cancer progression processes and miR-101 has been reported to suppress tumor proliferation, apoptosis, drug resistance, and metastasis by regulating several target genes [18]. Importantly, it was confirmed that miR-101 inhibited cell proliferation directly by inhibiting the expression of EZH2intransitional cell carcinomas, lung cancer, and embryonal rhabdomyosarcoma cells [19–21]. Moreover, forced miR-101 expression promoted HCC cell resistance to 5-FU by targeting EZH2 [22]. Furthermore, it was reported that miR-101 inhibited cell metastasis by down-regulating EZH2 expression in lung cancer and osteosarcoma [23, 24]. Thus, we predicted miR-101 as a promising biomarker in several cancers, which might offer a potential therapeutic target for clinical application. Here, we found that, miR-101 was downregulated in ESCC tissues, and reversed the tumor-promoting effects of PSMA3-AS1 in ESCC cells. EZH2 is a member of the polycomb repressive complex 2 (PRC2) and catalyzes the trimethylation of lysine 27 on histone 3 (H3K27me3) [25]. Forced EZH2 expression is frequently in a wide variety of cancers, including prostate cancer, ESCC, hepatocellular carcinoma, and lung cancer et al [26–29]. Dysregulation of EZH2 is implicated in ESCC cisplatin resistance, proliferation, invasion, and metastasis, and is associated with poor outcome in ESCC [30–34]. In this study, using various experiments, we confirmed that PSMA3-AS1 can promote the proliferation and metastasis of ESCC through the miR-101/EZH2 pathway.

## CONCLUSION

We found that PSMA3-AS1 is highly expressed in ESCC tissues and exerts its oncogenic effects via acting as a sponge of miR-101, thereby up-regulating EZH2 expression. The PSMA3-AS1/ miR-101/ EZH2 axis promotes ESCC progression and may serve as a potential promising therapeutic target of ESCC.

## MATERIALS AND METHODS

### Cells, clinical tissues, tissue microarrays, and immunohistochemistry

The human ESCC cell lines TE1, TE10, TE11, KYSE150, KYSE450, EC9706, and EC109 were purchased from the Shanghai Institute of Cell Biology, Chinese Academy of Sciences (Shanghai, China). All of the EGCC cell lines were cultured in Dulbecco’s modified Eagle’s medium (DMEM; Gibco, USA) with 10% FBS (Gibco, USA) and cultured at 37 °C with 5% CO_2_.

ESCC tissues and adjacent non-tumor tissues were obtained from 120 untreated patients undergoing primary surgical resection at the Cardiothoracic Surgery, The Second Affiliated Hospital of Nanchang University between January 2012 and August 2014. Tissues were snap-frozen in liquid nitrogen prior to RNA isolation and reverse transcription-quantitative polymerase chain reaction (RT-qPCR). Tissue microarray (TAM) and immunohistochemistry (IHC) staining analyses of EZH2 were performed as previously described [35]. This study was approved by the Human Ethics Committee of the Second Affiliated Hospital of Nanchang University and written informed consent was obtained from every patient.

### RT-qPCR

RT-qPCR was performed as described in a previous publication [36]. In brief, total RNA was extracted from ESCC cell lines, tumor tissues, and adjacent non-tumor tissues with a Trizol kits (Invitrogen, Carlsbad, CA, USA), and miRNA was extracted with a mirVanaTM miRNA kit (Ambion, Austin, TX, USA). U6 was used as an internal reference for miR-101 expression. GAPDH was used as an internal reference for PSMA-AS1 and EZH2 expression. The 2^−ΔΔCt^ method was adopted to calculate the relative levels of the target genes.

### RNA immunoprecipitation (RIP), luciferase reporter, and RNA pull-down assays

RIP and RNA pull-down assays were performed as described in previous study and in the supporting Methods section [37, 38].

### Western blotting, proliferation, clonal formation, wound healing and transwell migration assays

Western blotting, proliferation, clonal formation, wound healing and transwell migration assays were performed as described in our previous studies [39, 40].

### Cell transfection

The microRNA (miRNA) mimics and negative control utilized for transfection were purchased from GenePharma (Shanghai, China). PSMA-AS1 overexpression and short hairpin RNA adenovirus were constructed by GenePharma (Shanghai, China), and the transfection procedure was performed in accordance with the manufacturer’s protocol. The cells were harvested 72 h after transfection.

### Statistical analysis

Statistical analysis was performed as described in our previous studies [39, 40]. In brief, Student’s *t* test, correlation analysis, chi-square test, Kaplan-Meier’s analysis, and log-rank test results were generated using SPSS software (21.0; SPSS, Inc., Chicago, IL), and the diagrams were graphed using GraphPad Prism 7.0. P< 0.05 was considered statistically significant. All experiments were performed at three times.

### Ethics approval

Written consent was obtained from all participants, and the study was approved by the Ethics Committee of the Second Affiliated Hospital of Nanchang University.

## Supplementary Material

Supplementary Materials
