# Supplementary material for: Long non-coding RNA PSMA3-AS1 promotes malignant phenotypes of esophageal cancer by modulating the miR-101/EZH2 axis as a ceRNA
Source: Aging (Albany NY). 2020 Jan 31;12(2):1843–56. doi: 10.18632/aging.102716 (PMC7053621; doi:10.18632/aging.102716)
Supplement: Supplementary Materials [file aging-12-102716-s001..pdf]

SUPPLEMENTARY METHODS

RNA immunoprecipitation (RIP), luciferase reporter, and RNA pull-down assays

For RIP, the EZ-Magna RIP Kit (Millipore, USA) was used according to the product specification. In brief, cells were collected and lysed in complete RIP lysis buffer. Then, the cell extract was incubated with RIP buffer containing magnetic beads conjugated to a human anti-Ago2 antibody (Millipore, USA). Next, samples were incubated with proteinase K with shaking to digest proteins and the immunoprecipitated RNA was isolated. qRT-PCR was carried out to detect co-precipitated RNAs.

The sequence of EZH2 3′-UTR or PSMA-AS1 was amplified from ESCC cells cDNA. The potential miR-101 binding sites in EZH2 3′-UTR or PSMA-AS1 were mutated by the Quick-change site-directed mutagenesis kit (Agilent Technologies, Santa Clara, CA, USA). Then these sequences were respectively subcloned into pGL3 luciferase reporter vector (Promega, Madison, WI, USA). The wild-type/mutant EZH2 3′-UTR or PSMA-AS1 vector and control mimics or miR-101 mimics were co-transfected into ESCC cells. Then, the luciferase reporter assay system (Promega, Madison, WI, USA) was applied to examine the luciferase activity.

For RNA pull down assay, ESCC cells were transfected with biotinylated miR-101 or negative control (NC) (Genechem, Shanghai, China). Cell lysates were harvested 48 h after transfection and incubated with Dynabeads M-280 Streptavidin (Invitrogen, CA, USA) for 3 h at 4 °C according to the manufacturer’s protocol. The bound RNAs were purified using TRIzol according to the manufacturer’s protocol. qRT-PCR was carried out to detect purified RNAs.
